# Improved PVC/ZnO Nanocomposite Insulation for High Voltage and High Temperature Applications

**DOI:** 10.1038/s41598-023-31473-3

**Published:** 2023-05-04

**Authors:** Faiza, Abraiz Khattak, Ahmad Aziz Alahmadi, Hatsuo Ishida, Nasim Ullah

**Affiliations:** 1 School of Natural Sciences, National University of Sciences and Technology (NUST), Sector H-12, Islamabad, 44000 Pakistan; 2 Department of Electrical Power Engineering, National University of Science and Technology, USPCAS-E, Sector H-12, Islamabad, 44000 Pakistan; 3 Department of Macromolecular Science and Engineering, Case Western Reserve University, 10900 Euclid Avenue, Cleveland, OH 44106-7202 USA; 4 Department of Electrical Engineering, College of Engineering, Taif University, PO Box 11099, Taif, 21944 Saudi Arabia

**Keywords:** Chemical engineering, Electrical and electronic engineering

## Abstract

Nanosized inorganic oxides have the trends to improve many characteristics of solid polymer insulation. In this work, the characteristics of improved poly (vinyl chloride) (PVC)/ZnO are evaluated using 0, 2, 4 and 6 phr of ZnO nanoparticles dispersed in polymer matrix using internal mixer and finally compressed into circular disk with 80 mm diameter using compression molding technique. Dispersion properties are studied by scanning electron microscopy (SEM), Fourier transform infrared spectroscopy (FTIR), X-ray diffractometry (XRD), and optical microscopy (OM). The effect of filler on the electrical, optical, thermal, and dielectric properties of the PVC are also analyzed. Hydrophobicity of nano-composites is evaluated by measuring contact angle and recording hydrophobicity class using Swedish transmission research institute (STRI) classification method. Hydrophobic behavior decreases with the increase in filler content; contact angle increases up to 86°, and STRI class of HC3 for PZ4 is observed. Thermogravimetric analysis (TGA) and differential scanning calorimetry (DSC) are employed to evaluate the thermal properties of the samples. Also, continuous decrease of optical band gap energy from 4.04 eV for PZ0 to 2.57 eV for PZ6 is observed. In the meantime, an enhancement in the melting temperature, T_m_, is observed from 172 to 215 °C. To check the stability of materials against hydrothermal stresses, all the fabricated materials are then subjected to a hydrothermal aging process for 1000 h and their structural stability is analyzed using optical microscopy and FTIR analyses.

## Introduction

Various polymers having unique electrical, structural and physical properties are found in various applications in electrical power system. Many polymer systems have been studied to explore the properties and viability of different polymers like ethylene propylene diene monomer rubber (EPDM), silicone rubber (SiR), Nitrile butadiene rubber (NBR) polyamide (PA), polyethylene (PE) and poly(vinyl chloride) (PVC) to be used at commercial level for various applications. Depending upon the properties of polymers they can be used in many applications, for example memory device^1^, 3D printers^2^, adhesives^3^, lubricants^4^, membrane technology^5^, biomedical applications^6^, aerospace^7^, insulations^8^ and cosmetic industries^9^. Despite those advantageous properties, the polymer insulators lack stability against the environmental stresses. In particular, organic polymers show accelerated degradation when exposed to environmental stresses with moisture, pollutants, temperature, acid rain, leakage current, UV radiations and wind^3^. Application of composite materials often minimizes the adverse effect of degradation as the material used for reinforcement are stable under such environmental stresses. The combination of different materials imparts the characteristics from parent constituents without losing their identity^10^.

Recently, polymer nanocomposites are attracting attention of researchers, as a very small amount of nano-fillers greatly influences the properties of the polymers and enhance their structural stability against the environmental stresses^8^. For this purpose, various nanofillers are being investigated which include organic fillers like carbon nanotubes, graphene oxides, metal organic frame works (MOF’s), carbon dots, carbon nanoparticles and inorganic fillers, including metals, metal oxides such as silica, titania, and alumina etc., ceramics and glass^11,12^. In order to maintain the structural stability, it is evident from the literature that the addition of inorganic oxide nanofillers have shown remarkable contribution^13^. Among the polymeric materials used for cable insulation purposes, PVC is of utmost importance due to its versatile properties like high chemical and flame resistance, low cost and barrier properties^14^. Despite these advantages, the low surface energy of PVC limits its usage at industrial level. Furthermore, discoloration can take place by formation of conjugated bonds via dehydrochlorination at modest temperatures. The structural degradation of PVC can be initiated by environmental stresses when used for different electrical and thermal applications. In order to minimize the effect of environmental stresses, studies are being made to modify the properties of PVC by adding micro and nanofillers or by making blends with other polymers. Interestingly, with the addition of a small amount of nanofillers, significant enhancement in the properties could be seen^15^. However, to obtain maximum enhancement in properties, good dispersion must be achieved. As nanoparticles have strong tendency towards agglomeration due to their high surface energy, their incorporation must be carried out carefully to achieve maximum dispersion. Different methods are being used for achieving maximum dispersion, for example, melt blending, in situ polymerization and solution casting^16,16^. Melt blending technique showed the best results among all the other techniques being used in literature. Among nanoparticles inorganic oxide fillers are performing best as many metal, metal carbonates and metal oxides nanoparticles are being used in literature to tailor the properties according to the needs of applications. Inorganic fillers are of great interests due to their unique characteristics and stability against environmental stresses. Ramesan et al*.* investigated the effect of Cu-Al_2_O_3_ nanoparticles on the optical, dielectric and thermal properties of the PVC and polyethylene and observed pronounced effect for all the properties^18–21^. Many studies are reported on PVC/ZnO^22^, PVC/Al_2_O_3_^23^ and PVC/SiO_2_^24^ to investigate their different characteristics i.e. dielectric constant, contact angle, spreading coefficient, wetting energy and work of adhesion were investigated. To optimize electrical and dielectric properties, metal oxides are under consideration as they are more stable towards stresses, like heat, humidity and electric current. Their interaction with polymeric matrix enhance the stability of composite against different types of stresses^25^. Previously, Thabet et al. provided the comparison of different organic and inorganic fillers using clay, SiO_2,_ TiO_2_, ZnO and reported the effect of their size and concentration on PVC matrix where they found a profound effect of inorganic fillers on the dielectric, electric, optical and thermal properties of the polymer^26–28^. ZnO exhibits excellent electrical, thermal and optical properties, including its high dielectric constant, chemical stability, luminous transmittance and intensive UV and IR absorption. ZnO shows a strong interfacial interaction and it can enhance the thermal, mechanical and electrical characteristics of the polymer matrix and also provides the stability against environmental stresses^29^.

Considering relative ease of degradation of PVC with high humidity and temperature under environmental stress and taking advantage of the attractive properties of ZnO, this research proposes the fabrication of ZnO reinforced PVC nano-composite by using a melt blending technique to achieve maximum dispersion. The effect of variation of ZnO concentration on the optical, electrical and dielectric properties has been studied, especially in cable insulation and other outdoor applications. Also, the effect of humidity and temperature for 1000 h on the structure and surface of the polymer was evaluated.

## Experimental section

### Materials and methods

PVC having average molar mass of 48,000 g/mol and density 1.4 g/mL at 25 °C, ZnO nanoparticles of < 50 nm having density of 81.39 g/mol, 6% Al as dopant surface area 10.8 m^2^/g and > 97% purity, dioctyl phthalate (DOP) as plasticizer having specific gravity 0.9 and zinc stearate as heat stabilizer and melting point 128–130 °C were purchased from Sigma Aldrich, Germany.

### Instruments and conditions. Internal mixer

Internal mixer of Thermo Scientific (Haake Rheomix OS) attached with conductivity measurement unit Karlsruhe, GmbH model Typ005-150 (24 V DC, 0.49 A), Germany was used for mixing of polymer with ZnO nanoparticles.

#### Fourier transform infrared (FTIR) spectroscopy

Bruker Platinum ATR model Alpha, Ettlingen, Germany was used to perform FTIR analysis in the spectral range of 4000–500 cm^−1^. To obtain the absorbance against wavenumber, the sample was placed directly on a diamond ATR unit and the spectrum was ratioed against the background spectrum to obtain the absorbance mode.

#### X-ray diffraction (XRD) spectroscopy

XRD was performed with diffractometer of GmbH Germany model STOE and Cie at 40 kV having 2θ value ranges from 10° ≤ 2θ ≥ 80° with Cu Kα radiations with the wavelength of λ = 0.15406 nm for all the prepared nanocomposites.

#### Scanning electron microscopy (SEM)

To analyze the morphology of the prepared nanocomposites, field emission scanning electron microscope Zeiss FESEM model MIRA3 TESCAN Supra 55 VP, Jena, Germany was employed. Carbon coating was applied on an insulating sample surface to minimize the surface charge.

#### Optical microscopy

Optical microscopy was used at a 100 µm resolution and 20 × magnification. For this purpose advanced Metallurgical Microscopy MTI model EQ-MM500T was employed. It is evident from the literature that in short-term aging, only surface roughness takes place^30^. So, optical microscopy was used to find out changes or roughness on the surface during short term aging.

#### UV–VIS spectroscopy

Single beam UV visible spectrophotometer of PD instruments UK model T60, was used to check the absorbance and to calculate the band gaps of the samples.

#### STRI hydrophobicity analysis

The hydrophobicity classification of all the prepared samples was determined using standard images to distinguish one hydrophobicity class from the other, all the classes were noted at the time of experiment and images were taken for the record^31^.

#### Contact angle measurement

To measure the water contact angle, Video Contact Angle (VCA) of stationsweg model 28A, 2312 AV Leiden OPTIMA from ASTP Netherlands was used. Distilled water was filled into the motorized syringe and volume of the water droplet was maintained at 0.5 µL. The angle of the water droplet with the sample surface was measured. Only the advancing angle was measured and the reported value is the average of 5 samples.

#### Thermogravimetric analysis (TGA)

Thermogravimetric analysis was done using Mettler Toledo STAR System having 0.005% temperature accuracy. The fabricated samples were placed in an alumina holder and heated at 25 °C to 600 °C at the rate of 10 °C/min under inert environment with nitrogen purging at 10 mL/min.

#### Differential scanning calorimetry (DSC)

DSC analysis was performed using TA instruments model 2920 using aluminum pan as reference at a heating rate of 10 °C/min in temperature range of 24 °C and 300 °C. The instrument was purged by nitrogen gas at a rate of 10 mL/min.

### Fabrication of samples

The as-purchased PVC was first masticated in an internal mixer at 50 °C for 2 min. After that DOP, zinc stearate and nano-ZnO were added and melt blending was carried out for 3 min at 50 °C. Subsequently, the mixed material was compression molded at 120 °C for 5 min at 9 MPa and a circular disc of 80 mm diameter was finally fabricated.

**Figure 1:**
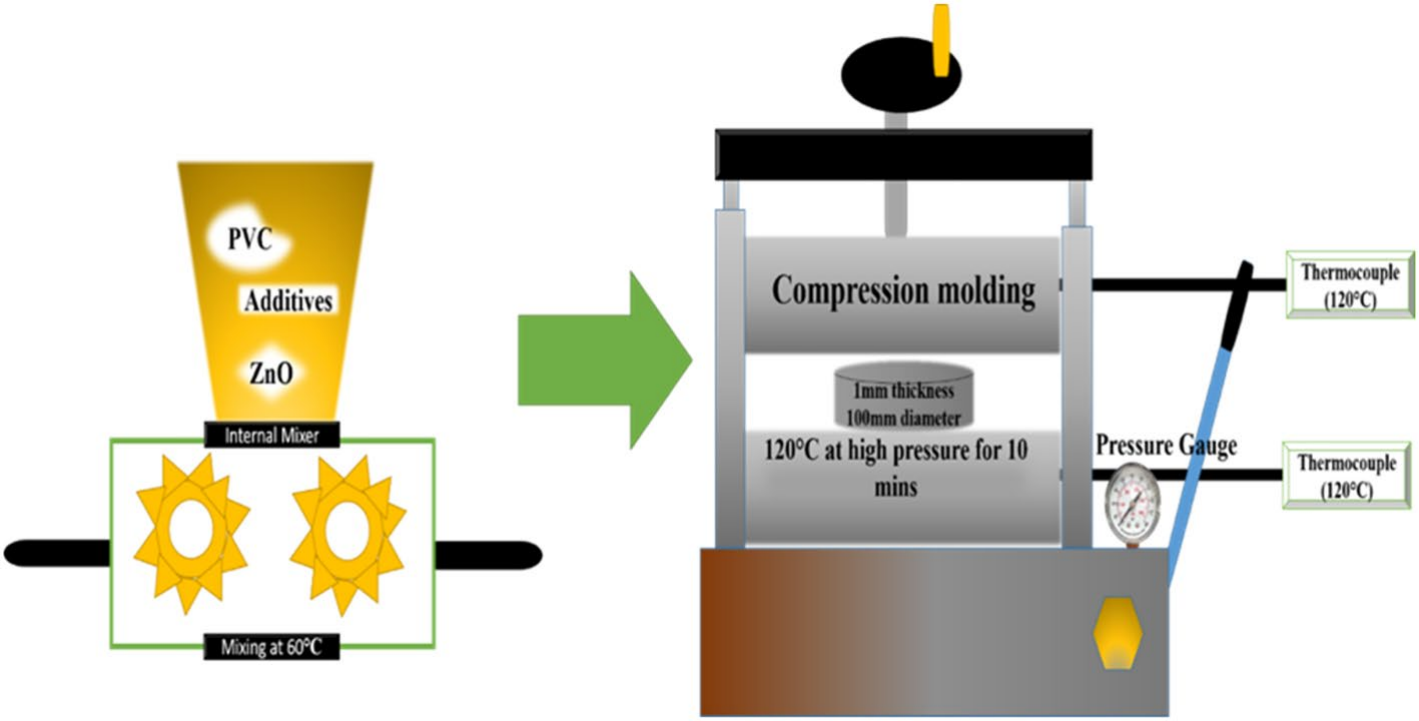
Schematic diagram for sample fabrication.

**Figure 2:**
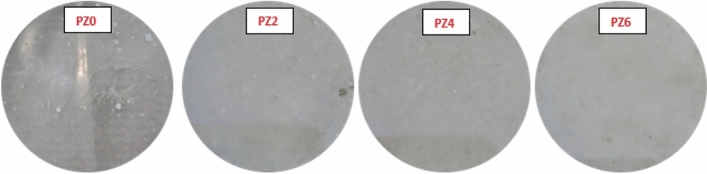
Prepared samples with their respective codes.

A schematic diagram of the sample fabrication is given in Fig. 1. Using this method, the different sample of 0, 2, 4 and 6 phr of ZnO were fabricated according to the compositions and their sample codes given in Table 1. The images of the prepared samples are given in Fig. 2.

**Table 1:** Composition and abbreviation of all the prepared samples.

Sr. #	PVC (phr)	ZO (phr)	DOP (phr)	Zinc stearate (phr)	Sample code
1	100	0	35	2	PZ0
2	100	2	35	2	PZ2
3	100	4	35	2	PZ4
4	100	6	35	2	PZ6

### Hydrothermal aging setup

**Figure 3:**
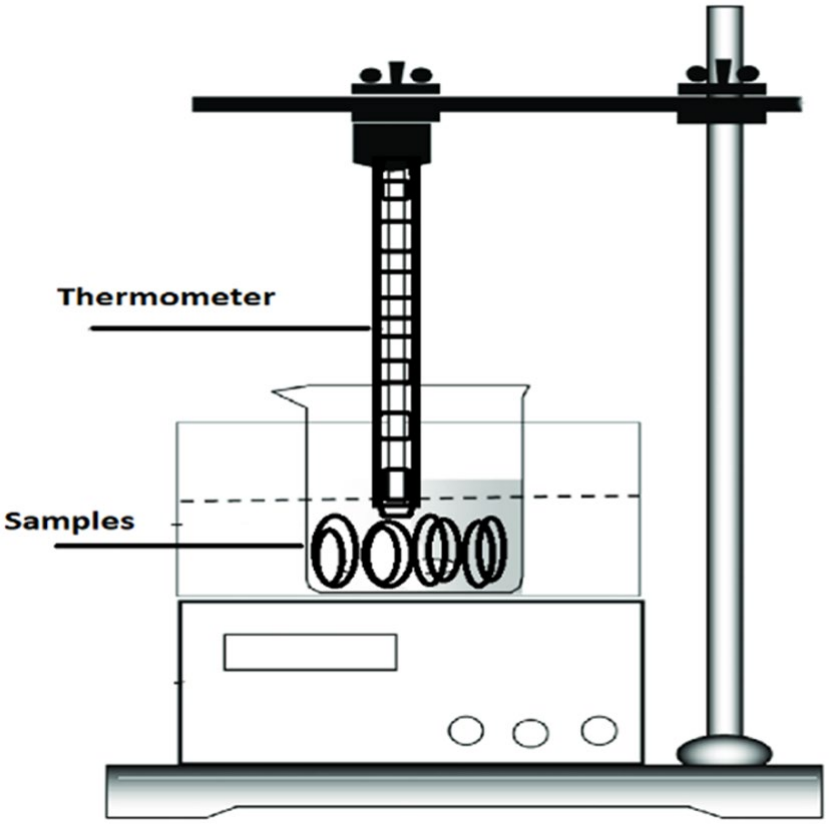
Schematic diagram of hydrothermal aging setup.

Hydrothermal stresses were applied using glass beaker filled with distilled water placed on hot plate maintaining temperature between 50 to 55 °C and all the fabricated sample were labelled and placed in the beaker for 1000 h. The structural and thermal stability of all the prepared samples were analyzed before and after the aging. Figure 3 shows the schematic diagram for aging setup.

## Results and discussion

### Fourier transform infrared spectroscopy (FTIR)

**Figure 4:**
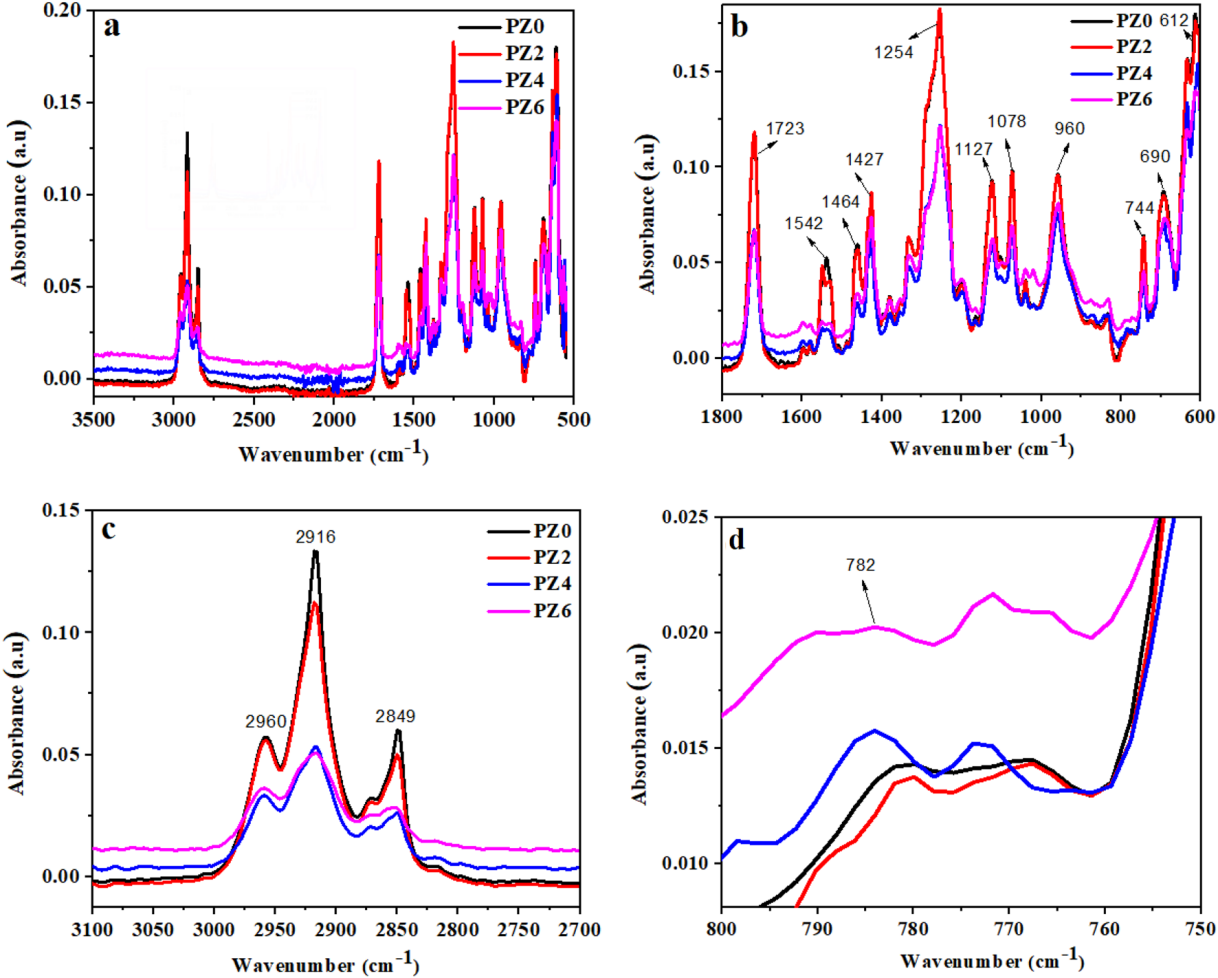
(**a**) FTIR Spectra of all the prepared samples. (**b**) FTIR Spectra of all the prepared samples in range 600–1800 cm^−1^. (**c**) FTIR Spectra of all the prepared samples in range 2700–3100 cm^−1^. (**d**) FTIR Spectra of all the prepared samples in the range 750–800 cm^−1^.

FTIR analysis was employed to confirm the presence of polymer matrix and other additives. Comparison of FTIR spectra of all the prepared samples is given in Fig. 4a.

The FTIR absorbance bands of PVC could be divided into three regions. Those are numerous C-H modes that appear in the region of 2970–2800 and 1550–1250 cm^−1^, C–C stretching that ranges from 1200 to 900 cm^−1^, and the C–Cl region that ranges^31^ from 700 to 600 cm^−1^. The characteristic band for PVC could be seen in Fig. 4b 2960, 2916 and 2849 cm^−1^ which correspond to the CH stretching from CH_2_ and CH–Cl. Bands at 1542, 1464 and 1427 cm^−1^ are due to the CH_2_ wagging and at 1254 cm^−1^ is for CH in-plane deformation of CH–Cl as shown in Fig. 4c. Some other bands at 1127 cm^−1^ and 1078 cm^−1^ are due to the C–C stretch, 960 cm^−1^ for the CH_2_ rocking and 744 cm^−1^, 690 cm^−1^ and 612 cm^−1^ are the C–Cl stretching.

The band near 782 cm^−1^ corresponds to the ZnO stretching vibration^32^. This band was not seen in case of neat PVC and with the increase in the concentration of ZnO, the absorbance value also increased as shown in Fig. 4d. Although the band intensity is not significant due to higher intensities of PVC bonds, the results were nonetheless confirmed by comparing it with the XRD diffraction peaks. A band at 1723 cm^−1^ appeared from the carbonyl group of zinc stearate^31^.

### X-ray diffraction spectroscopy (XRD)

**Figure 5:**
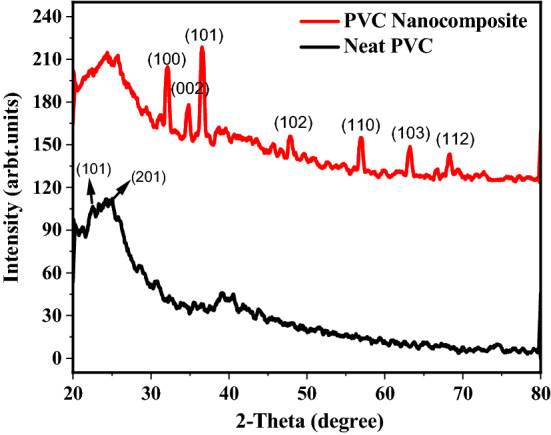
X-ray diffraction comparison of neat PVC and PVC nanocomposite.

X-ray diffraction spectroscopy was employed to confirm the successful incorporation of nano-ZnO into the polymer matrix. As shown in Fig. 5, all the ZnO peaks of hexagonal wurtzite crystal structure are observed in the spectra of nanocomposite at 2-θ values at 32°(100), 35°(002), 38°(101), 48°(102), 56°(110), 63°(103) and 68°(112) that are consistent with JCPDS 75-0576 having lattice parameters of *a* = 0.325 nm and *c* = 0.521 nm.

All the ZnO peaks located between 30° to 75° and a broad peak of PVC appeared between 20° to 30° (JCPDS 15-09999) that indicates the amorphous nature of PVC.

### Scanning electron microscopy

**Figure 6:**
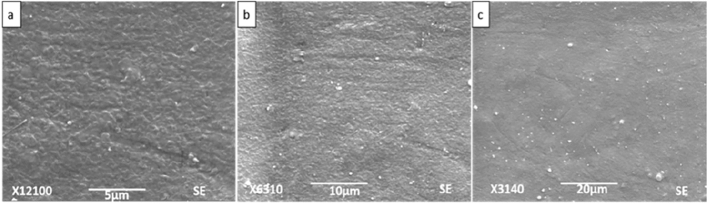
SEM images of PZ0 at (**a**) 5 µm, (**b**) 10 µm, (**c**) 20 µm.

**Figure 7:**
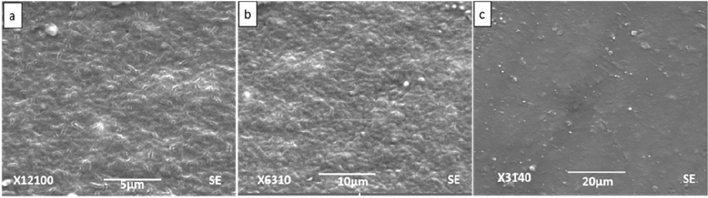
SEM images of PZ2 at (**a**) 5 µm, (**b**) 10 µm, (**c**) 20 µm.

**Figure 8:**
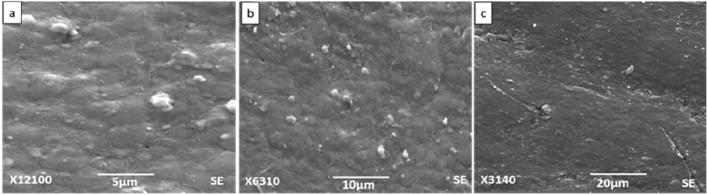
SEM images of PZ4 at (**a**) 5 µm, (**b**) 10 µm, (**c**) 20 µm.

**Figure 9:**
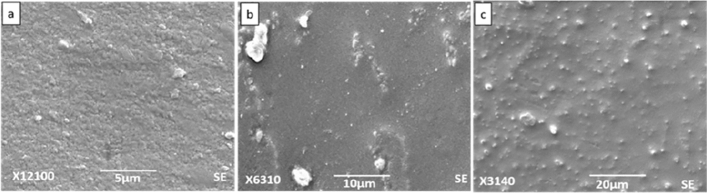
SEM images of PZ6 at (**a**) 5 µm, (**b**) 10 µm, (**c**) 20 µm.

Figures 6, 7, 8 and 9 show SEM images of all the nanocomposite at 5, 10 and 20 µm. It is clear from the images that surface roughness and brittleness increased by increasing amount of filler into the PVC matrix and it was due to the agglomeration of nanoparticles in the samples^33^. It can be seen from Fig. 7 that PZ2 showed maximum dispersion with minimum possible distance. As the filler contents increased, i.e. for samples PZ4 and PZ6, agglomeration caused the brittleness of the sample and this effect maximized for the sample PZ6.

### UV–VIS spectroscopy

**Figure 10:**
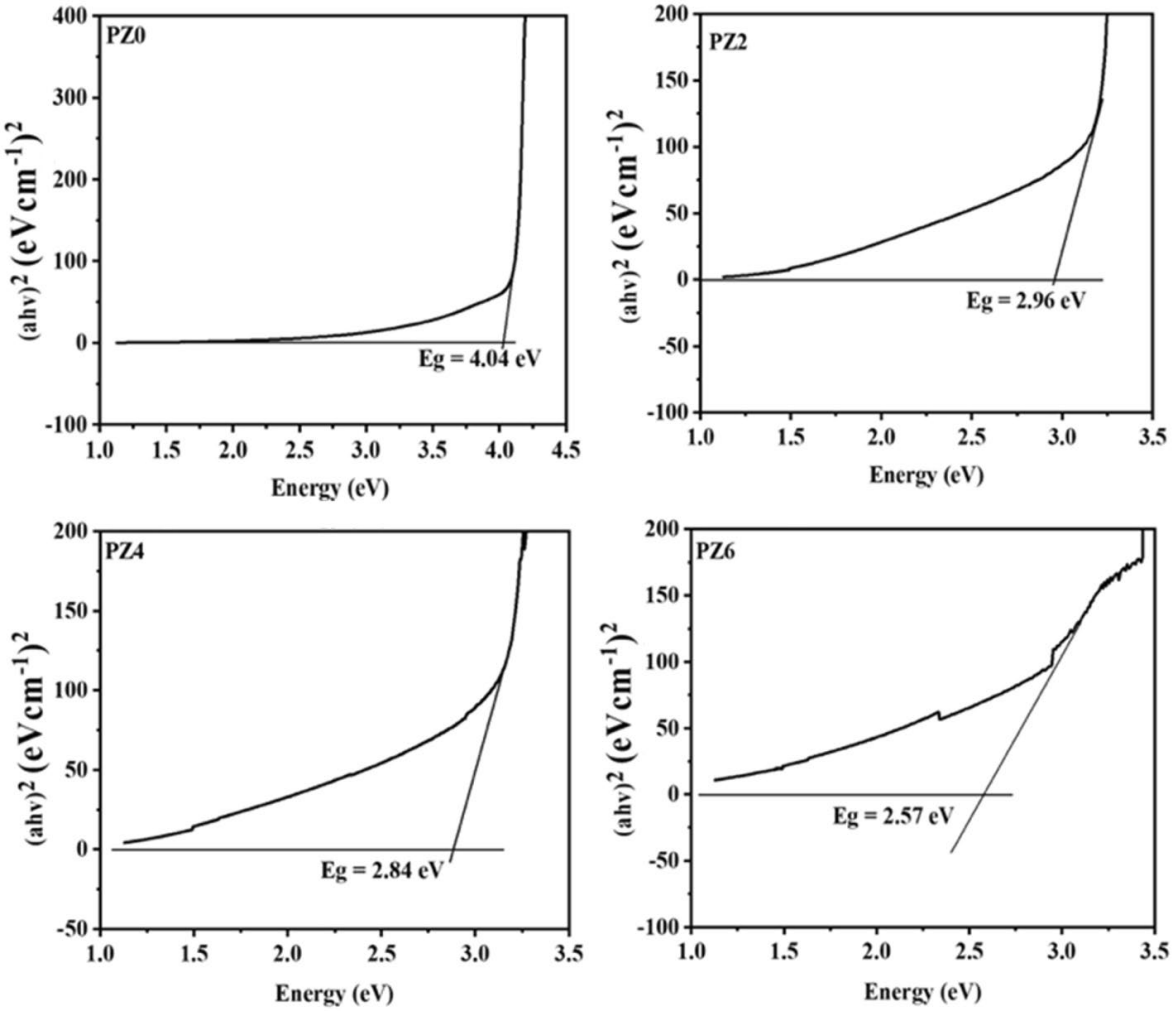
Band gap calculation of all the prepared samples.

The effect of ZnO nano-filler on the band gap of PVC was investigated by plotting Tauc plots between absorbance coefficient (ahν)^2^ at y-axis and energy (hν) at x-axis and then by extrapolating a straight line towards x-axis where value of y-axis = 0 for all the prepared composites. It is reported in the literature that PVC shows band gap value around 4 eV. and it can be observed from Fig. 10 that the band gap value obtained for PZ0 was 4.04 eV and it decreased to 2.96, 2.84 and 2.57 eV as concentration of ZnO increased to 2 phr, 4 phr and 6 phr respectively. The decrease observed in the band gap is due to the trapping centers, formed due to the addition of ZnO which helped the recombination of the electrons^34^.

### Hydrophobicity

The interaction of material surface and water is considered as most important phenomena to weaken the mechanical and electrical properties of insulators. Therefore, it is of utmost importance to check the hydrophobic behavior of an insulator, which is discussed in the following section.

#### STRI hydrophobicity classification

**Figure 11:**
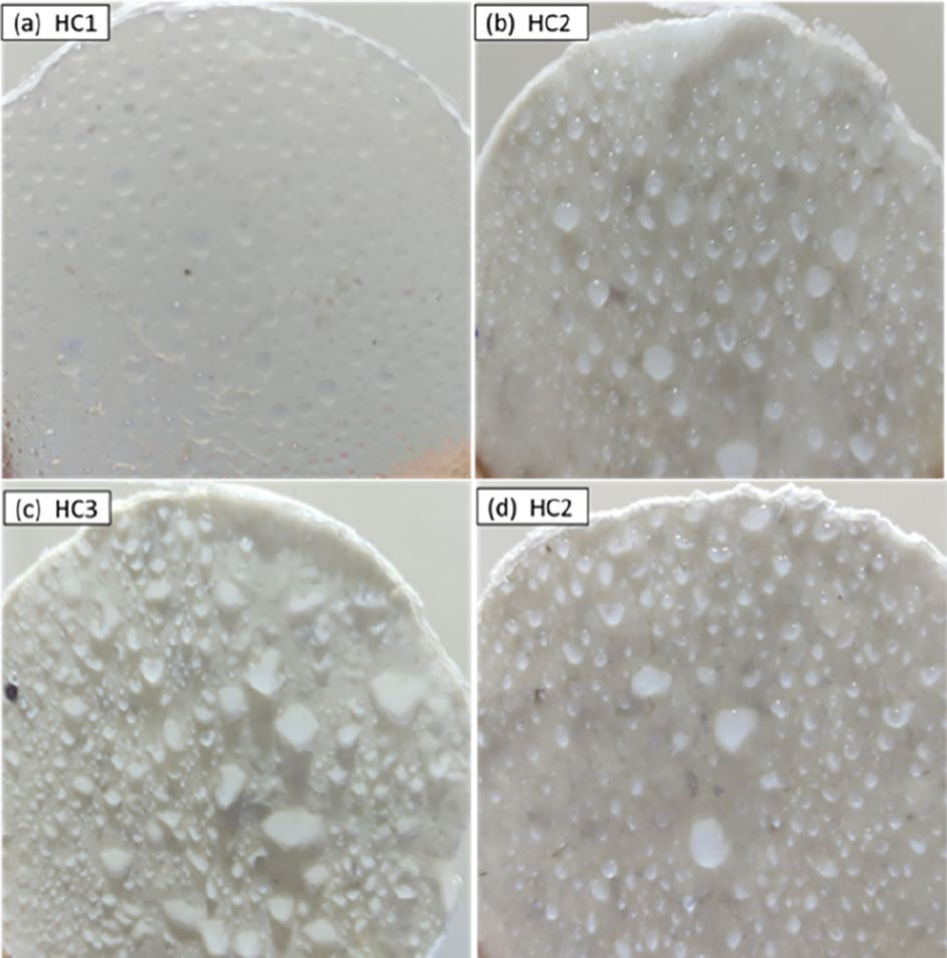
STRI hydrophobicity classification of (**a**) PZ0, (**b**) PZ2, (**c**) PZ4, (**d**) PZ6.

The Swedish Transmission Research Institute (STRI) hydrophobicity classification was employed to classify the class of hydrophobicity as shown in Fig. 11. The images are compared with the HC1–HC6 classification guide of STRI and are given in Fig. 11. In the STRI classification, HC1 is considered the most hydrophobic. The loss in hydrophobicity of PVC after addition of nano-ZnO is observed. HC1 class is seen in case of PZ0 which turns into HC2 and HC3 for PZ2 and PZ4, respectively. The loss in hydrophobicity is observed due to the hydrophilic nature of zinc oxide surface which interacts with water. However, PZ6 shows HC2 class whose increase in hydrophobic character is due to the loss of available ZnO surface caused by the agglomeration of zinc oxide nanoparticles. It is further explained in contact angle measurements shown below where detailed hydrophobicity analysis is described^35^.

#### Contact angle measurements

**Figure 12:**
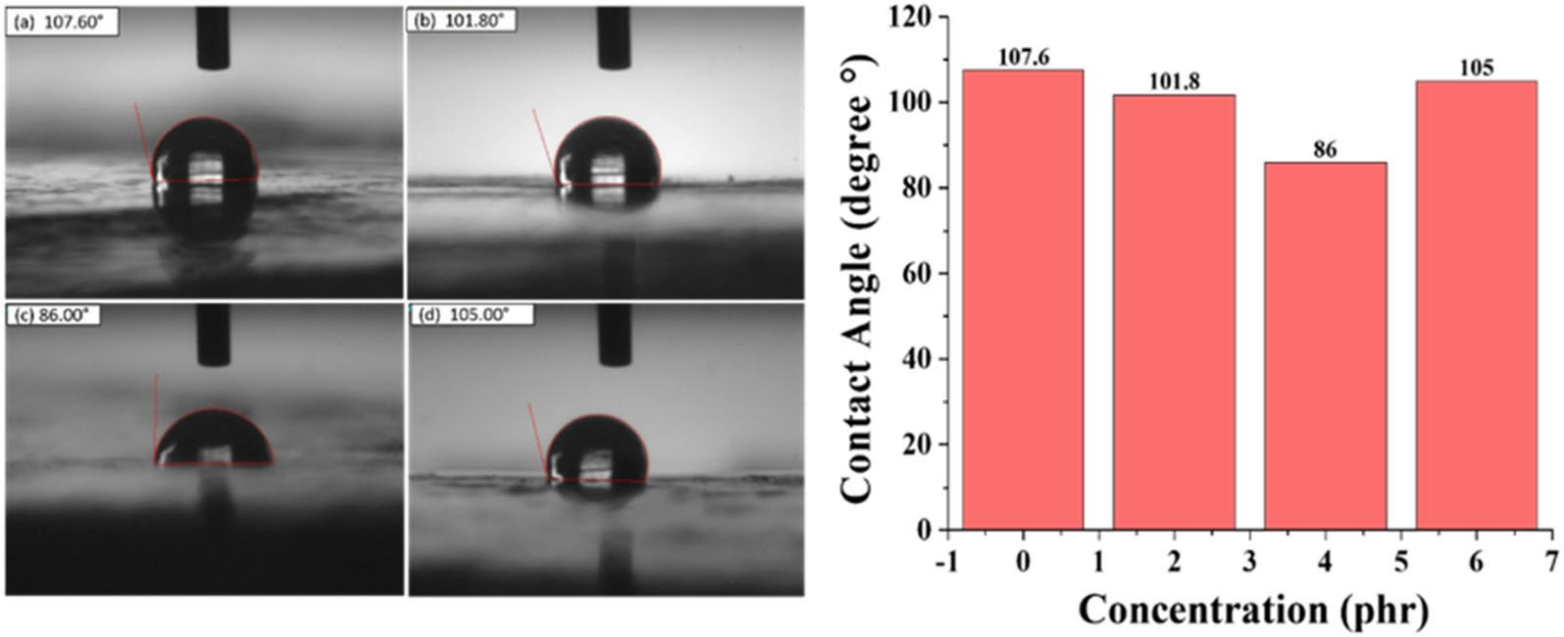
Contact angle measurement of (**a**) PZ0, (**b**) PZ2, (**c**) PZ4, (**d**) PZ6.

In order to perform detailed quantitative analysis to check the hydrophobic nature of PVC and its composites, contact angle measurements were done. The greater the contact angle, the more the hydrophobic nature of the composite^36^. It is evident from Fig. 12 that contact angle decreased from 107.6° to 101.8° and 86° as concentration of ZnO increased from 0 to 2 and 4 phr. This decrease in the contact angle is due to the interaction of ZnO nanoparticles with the water molecule as described above. With the increase in ZnO concentration, the number of ZnO nanoparticles increases on the surface of PVC disc which leads to the increase in hydrophilicity. Upon further increase in ZnO concentration to 6 phr the contact angle slightly decreases from the 4 phr sample of 107.6° to 105°, which is consistent with the STRI classification changing towards more hydrophilic. As the nanoparticle distance reduces due to the increased concentration, attraction by the van der Waals forces increases, leading to agglomeration. At higher concentrations, agglomeration cannot be avoided completely, thus the increase in contact angle in the case of PZ6^35^.

### Thermogravimetric analysis (TGA)

**Figure 13:**
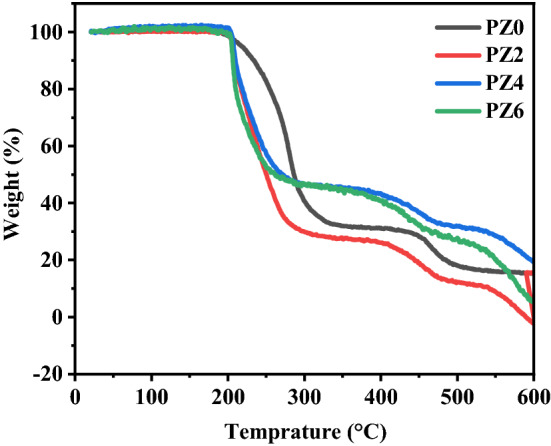
TGA thermograph of all the prepared samples.

**Figure 14:**
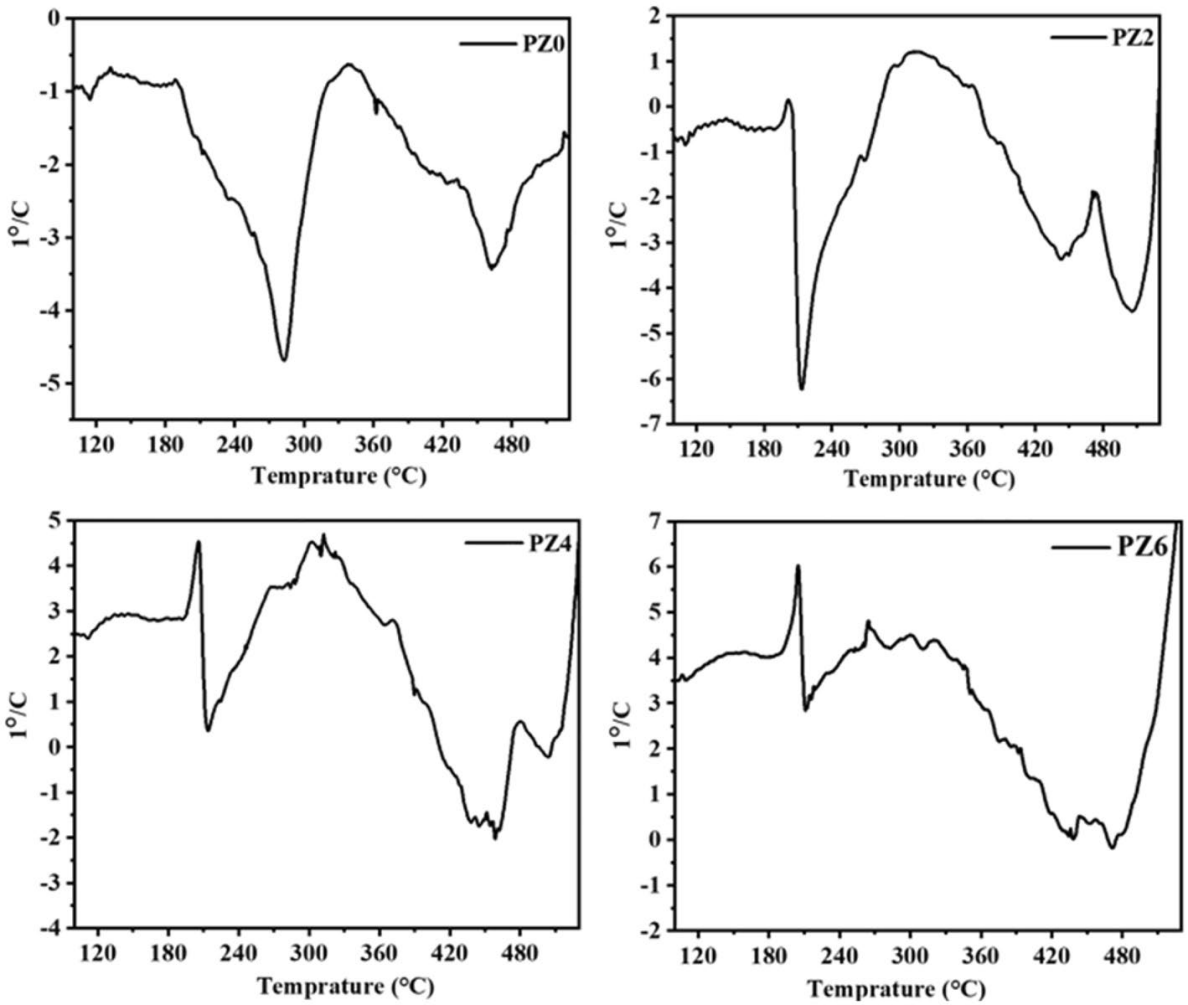
DTA thermograph of all the prepared samples.

Thermogravimetric analysis was employed to study the thermal stability of PVC and its composites. Figures 13 and 14 show the TGA and differential thermal analysis (DTA) thermograms of all the prepared samples, respectively. It is evident that in case of neat PVC two temperature regions of weight loss appeared. First region started around 200 °C that can be attributed to the dehydrochlorination which leads to the formation of conjugated polyene sequences, leading to discoloration. No weight loss before 200 °C occurred in case of neat PVC which confirms the absence of any moisture and solvent. Second weight loss region for neat PVC can be seen around 450 °C which appeared due to the thermal cracking of carbonaceous conjugated chains^37^. From Fig. 13, it is evident that that PV0 was stable up to 450 °C; however, nanocomposites were stable up to 500 °C.

In case of PVC-ZnO nano-composite three weight loss regions were observed where the first two were the same as for PVC However, the third weight loss region can be seen around 520 °C whose magnitude corresponds to the char yield which was 11, 24, and 30% for PZ2, PZ4 and PZ6, respectively^38^. The first region of weight loss started earlier than the neat PVC which means that ZnO nanoparticles showed the catalytic activity for dehydrochlorination of PVC chains^39^. Different regions of weight loss could be seen in DTA graph in Fig. 14.

### Differential scanning calorimetric (DSC) analysis

**Figure 15:**
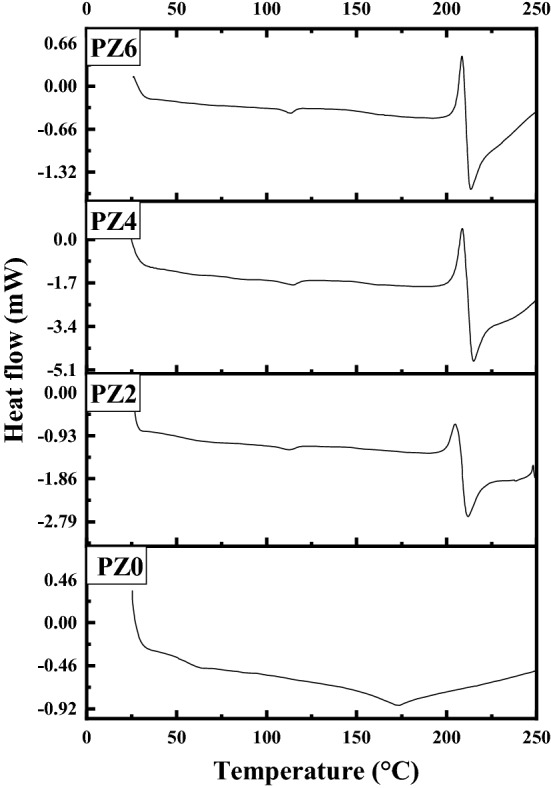
DSC thermograph of all the prepared samples.

**Figure 16:**
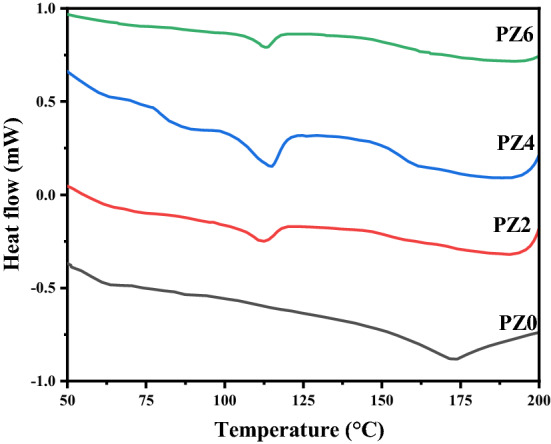
Comparison of water evaporation from nano-composite vs. neat PVC.

DSC analysis is performed to study the thermal properties of the polymer in the temperature range from 24 to 250 °C. The comparison of DSC thermograms for all the prepared samples is given in Fig. 15. It is evident from the thermograms that an exothermic process is seen around 205 °C in case of nanocomposites that was not observed for neat PVC. The first endotherm corresponds to the water evaporation from the surface of the ZnO hydrophilic nanoparticles and it is seen from Fig. 16 that the water evaporation takes place only in case of nanocomposite and the endotherm is missing in neat PVC due to the absence of ZnO.

**Figure 17:**
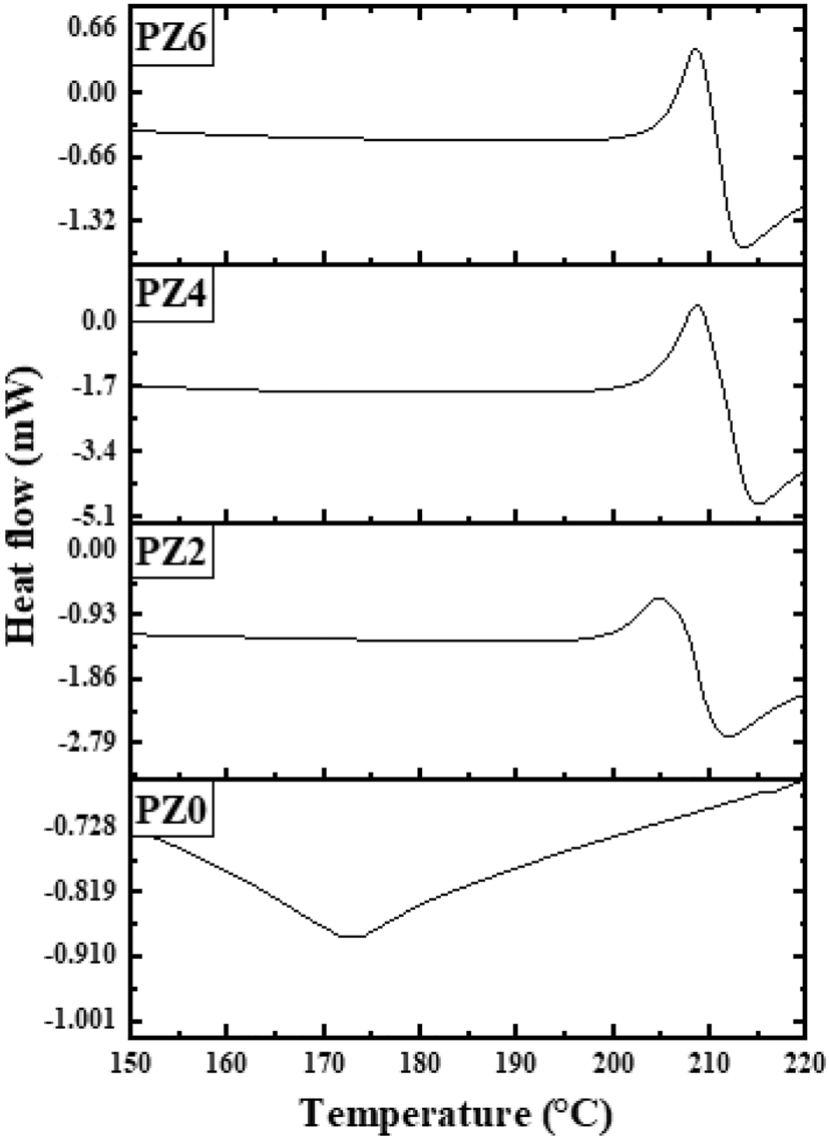
DSC thermograph in temperature range 175–300 °C.

After that an endothermic melting peak can be observed in the case of PZ0 at 172 °C. However, an exothermic crystallization is seen just before the melting endotherm in case of all the composites at 208 °C. The melting peak of all the composite shifted from 172 to 215 °C for all the composite as shown in Fig. 17. The shift of melting endotherm might be due to the nucleation process of nanofiller or the crystal quality formed in between the nanofiller spaces is better due to the stress field by ZnO nano-filler. It is also possible that the crystals are confined between narrow spaces of the ZnO nanoparticles and confined crystals are known to exhibit higher melting endotherm temperature^40^. Further detailed study is needed before concluding the molecular reasons for this behavior.

## Effect of hydrothermal conditions on structure and surface

**Figure 18:**
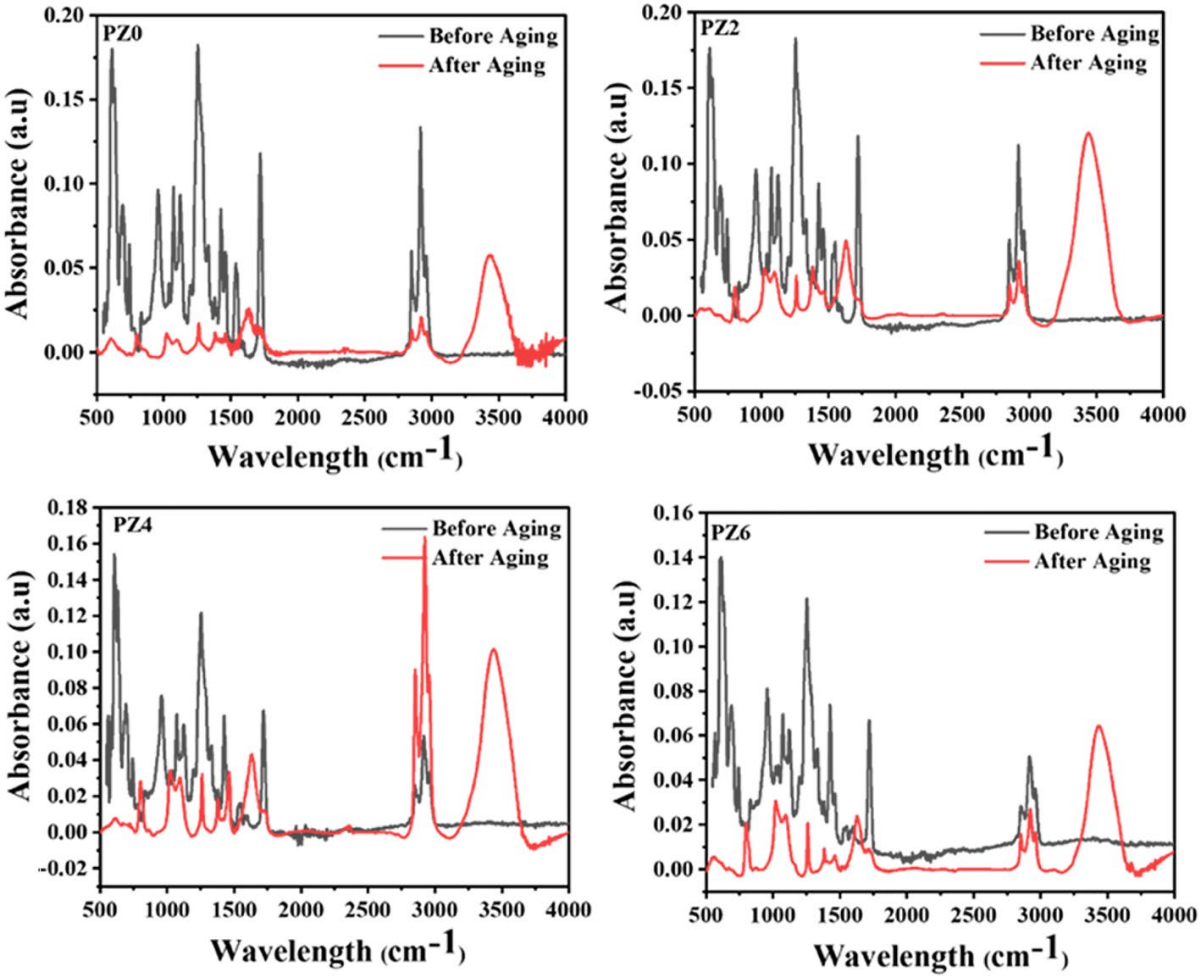
FTIR spectra of all the prepared samples before and after aging.

FTIR and Optical microscopy were employed to investigate the structural stability of the material against applied hydrothermal conditions. The comparison of absorption peaks of all the prepared samples is given in Fig. 18. Due to the lack of reliable internal thickness band, the results presented should be considered at best semi-quantitative. The absorbance intensities for different vibrational modes are compared in Table 2 and it is clear that decrease is maximum for PZ0; however, by adding ZnO filler in the PVC matrix the structural stability enhanced.

**Table 2:** Percentage degradation of all the prepared samples after aging.

Wavenumber (cm^−1^)	Vibrational mode	Absorbance (a.u)	
		Before aging	After aging
PZ0			
2796–3053	CH stretching from CH–Cl and CH_2_	0.13	0.02
1325–1536	CH_2_ wagging	0.08	0.01
917–1211	C–C stretch	0.09	0.01
736–890	CH_2_ rocking	0.09	0.01
PZ2			
2796–3053	CH stretching from CH–Cl and CH_2_	0.11	0.03
1325–1536	CH_2_ wagging	0.08	0.03
917–1211	C–C stretch	0.09	0.03
736–890	CH_2_ rocking	0.09	0.03
PZ4			
2796–3053	CH stretching from CH_2_	0.05	0.15
1325–1536	CH_2_ wagging	0.06	0.03
917–1211	C–C stretch	0.07	0.03
736–890	CH_2_ rocking	0.04	0.02
PZ6			
2796–3053	CH stretching from CH–Cl and CH_2_	0.05	0.02
1325–1536	CH_2_ wagging	0.07	0.01
917–1211	C–C stretch	0.06	0.03
736–890	CH_2_ rocking	0.04	0.02

The absorbance in the region of 1536–1325 cm^−1^ for C–C stretching indicates the decrease from 0.09 to 0.01 for PZ0, 0.09 to 0.03 for PZ2 and 0.04 to 0.02 for PZ4 and 0.06 to 0.03 for PZ6. Similarly the absorbance intensities after aging for all of the bonds decreases due to structural degradation of the bonds. Absorbance intensities of different vibrational modes are given in Table 2. It is evident that maximum decrease in the intensities is observed for PZ0 however in case of the PZ2, PZ4 and PZ6 the decrease was minimized as compare to the neat PVC.

**Figure 19:**
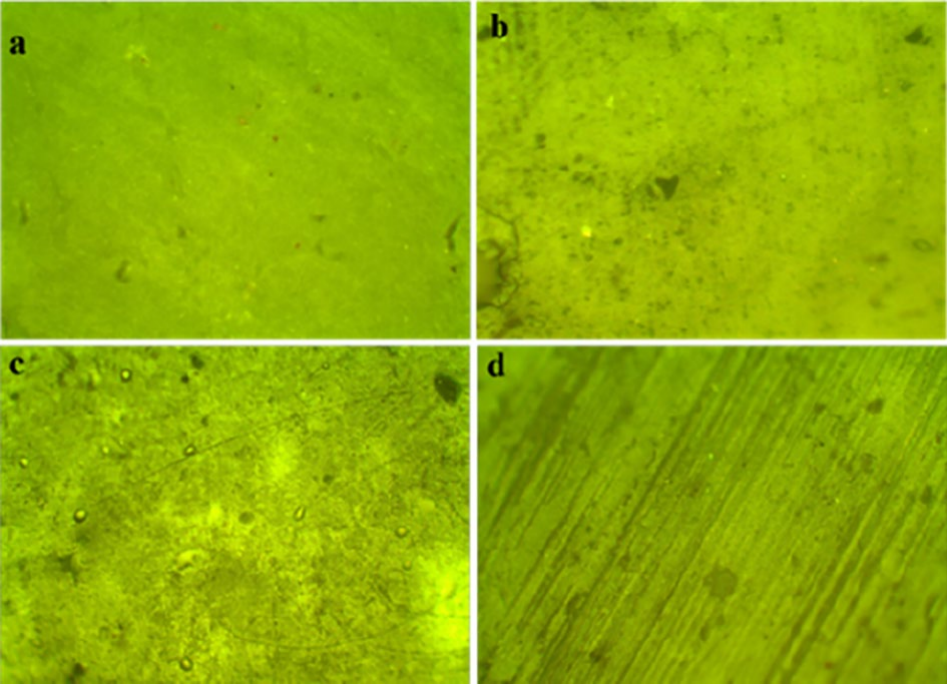
Optical microscopy images of (**a**) PZ0, (**b**) PZ2, (**c**) PZ4, (**d**) PZ6 before aging.

**Figure 20:**
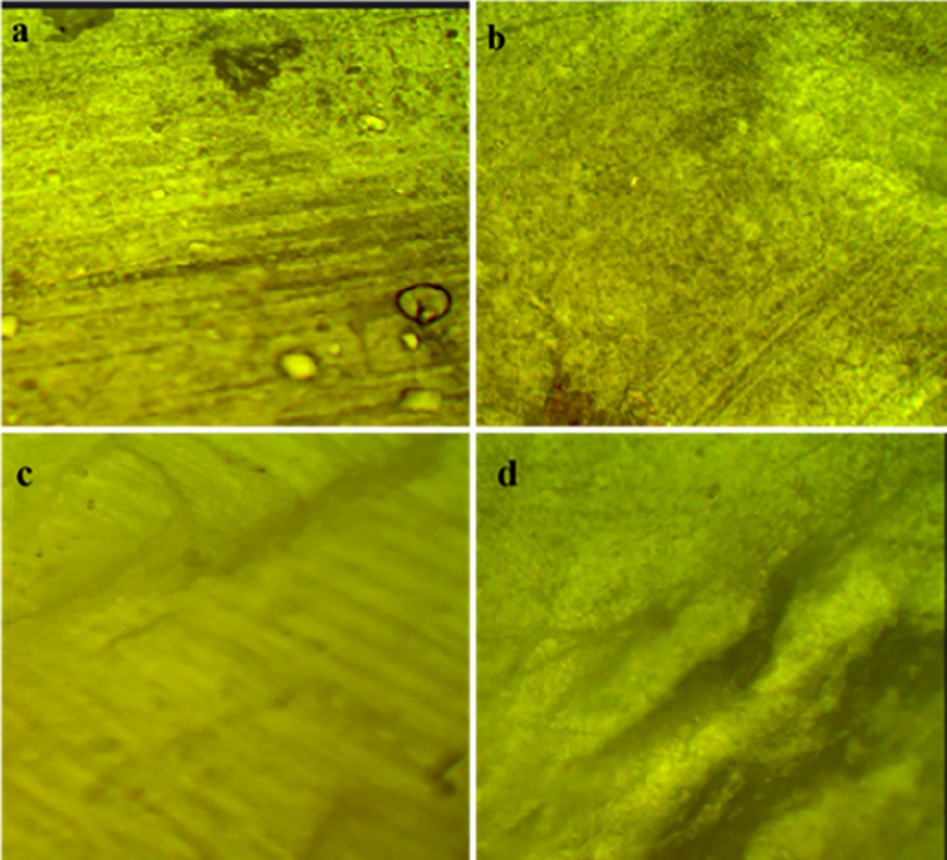
Optical microscopy images of (**a**) PZ0, (**b**) PZ2, (**c**) PZ4, (**d**) PZ6 after aging.

Optical microscopy was employed to analyze the surface topology of the PVC and nanocomposite before and after aging to check the change in surface under hydrothermal conditions. It is found from Fig. 19 that with the addition of ZnO in the polymer matrix the surface roughness was enhanced^22^. Figure 20 shows the images of all the samples after aging. There are visible cracks on the surface of PZ0 after 1000 h of aging; however, the PZ2, PZ4 and PZ6 the surface integrity better.

## Comparison section

Comparison of previously reported PVC based various nano-composites is given in Table 3. From previously reported studies it is observed that maximum dispersion achieved up to 6% of the nano-filler into the polymer matrix and further increase in concentration caused agglomeration, however in our method, highly sensitive internal mixer was used to achieve maximum dispersion. That resulted into maximum dispersion of filler up to 4% in addition with other additives and stabilizers. All the previous studies report the dispersion of filler only, hence this method showed maximum dispersion of the filler into polymer in comparison to any other method reported up till now. Also in previous studies most of the preparation techniques involves solution casting method which involves the use of organic solvent which in not an environmental friendly technique, on the other hand current research involves solventless fabrication. From the earlier research it was observed that different type of fillers exhibit pronounced effect on the various properties of polymers, however ZnO and TiO_2_ proved to be most efficient fillers to be used in PVC depending upon their stability against the stresses. All the inorganic nano-filler enhance the surface roughness, but the current research involves the addition of DOP as stabilizer which help to retain the surface smoothness to some extent. There are very limited studies are reported for PVC/ nano-composite degradation, also previously no study is reported to investigate the effect of hydrothermal stresses on the PVC/ZnO nano-composite that are most crucial stresses that needs to be investigate that will help to improve economical situation in PVC industry.

**Table 3:** Comparison of various pvc based nanocomposites.

Ref. no.	Polymer nano-composite	Concentration prepared (wt %)	Key findings
^22^	PVC/ZnO	2.5, 5, 10 (annealed, nonannealed)	Optical results were studied and effect of concentration was determined and it was seen that band gap decreases with increase in concentration
^33^	PVC/ZnO	0, 2, 4, 6, 8	The adequate dispersion of filler up to 6% was observed. However agglomeration on further increase in concentration was observe. Also profound effect on storage modulus with increase in concentration was observed
^38^	PVC/ZnO	Neat, 0.62 g in 15 wt% of PVC	The decrease in hydrophobic behavior by adding nano-filler was observed
^37^	PVC/ZnO	2 wt% ZnO in different mol.wt of PVC (P60, P90, P130)	The catalytic effect for the loss of HCl in first step of TGA was observed
^41^	PVC/ SiO_2_	No weight % of nano-filler is given	The effect of coupling agent to enhance the interaction of polymer and nanofiller was evaluated and it was observed that mechanical properties were improved with enhanced interaction of polymer and filler
^42^	PVC/SiO_2_	1, 2, 4	The cracks and rough surface was observed by adding nanofiller and increase in band gap was seen
^43^	PVC/Al_2_O_3_	0, 2, 4, 6	The fine dispersion of nano-filter into the polymer was observed and decrease in bad gap values with increase in nano-filler concentration was seen
^44^	PVC/TiO_2_	3 phr	The effect of nano-filler on mechanical properties before and after UV-aging was evaluated and it was observed that incorporation of nano-filler provides resistance against degradation under UV exposure

## Conclusion

To enhance the thermal, optical, electrical and dielectric properties of poly(vinyl chloride), nanopowder of ZnO was used as filler. Varying concentration of ZnO (2, 4 and 6 phr) were melt mixed using highly sensitive internal mixer to achieve maximum dispersion of filler into the polymer matrix. It was concluded that PZ2 showed the maximum dispersion; however further increase in concentration resulted in the agglomeration of filler. The loss of hydrophobic behavior is observed initially by adding nano-filler for both contact angle and STRI classification. The contact angle decreased from 107.6° to 101.8° for PZ0 and PZ2 respectively. However reverse trend was seen when concentration was further increased to 4 and 6 phr due to agglomeration. Nanocomposites were stable up to 220 °C and it was observed that filler is acting as catalyst which accelerates the dehydrochlorination reaction for the PVC matrix and the char yield changed from 11 to 24% and 30% for the PZ2, PZ4 and PZ6 at 500 °C, respectively. It was observed from DSC thermograms that T_m_ of PVC shifted from 175 °C to 215 °C for PZ2, PZ4 and PZ6. The band gap decreased from 4.04 to 2.96, 2.84 and 2.57 eV for PZ2, PZ4 and PZ6 respectively. By varying the ZnO concentration the properties of the PVC can be tuned according to desired application. It was seen that the degradation evaluated from the FTIR absorbance intensities was maximum in case of PZ0. However, after aging, the decrease in absorbance intensities was less in case of nanofiller incorporated polymer. The aging studies suggest that the incorporation of ZnO filler enhances the stiffness and structural stability of polymer to retain their structure against the hydrothermal conditions. Further long-term aging studies after regular interval are required to check the maximum endurance of the material against the hydrothermal conditions.

## Data Availability

The data presented in this study are available on the request from the corresponding author.
